# Supplementary material for: MetaRibo-Seq measures translation in microbiomes
Source: Nat Commun. 2020 Jun 29;11:3268. doi: 10.1038/s41467-020-17081-z (PMC7324362; doi:10.1038/s41467-020-17081-z)
Supplement: Supplementary file 10 — Supplementary Data 7 [file 41467_2020_17081_MOESM10_ESM.zip › File2/Confidence_High_Taxonomy/345856_out.krona.html]

Javascript must be enabled to view this page.

members
magnitude
magnitudeUnassigned
count
unassigned
taxon
rank

345856\_out

36

2
superkingdom
36

36
1239
phylum

186801
class
36

order
36
186802

SRS042628\_contig\_number\_32551SRS057717\_contig\_number\_6771SRS064645\_contig\_number\_contig-100\_1695.1695SRS077552\_contig\_number\_22634SRS149879\_contig\_number\_13440SRS893252\_contig\_number\_8439
6

29
family

SRS014923\_contig\_number\_38662SRS015794\_contig\_number\_7046SRS098717\_contig\_number\_14208SRS142599\_contig\_number\_30901SRS142890\_contig\_number\_31736
5
186803

1

SRS144135\_contig\_number\_3358
1952136
species

species
665950

SRS057478\_contig\_number\_3533SRS143991\_contig\_number\_15860
2

1

SRS063518\_contig\_number\_22093
1952133
species


SRS013940\_contig\_number\_19127SRS014459\_contig\_number\_11672SRS148253\_contig\_number\_9199
3
species
1898203

4
genus
841

1897017
species
1

SRS049773\_contig\_number\_23278

species
2293126

SRS1055067\_contig\_number\_14464
1


SRS148091\_contig\_number\_9666
1
species
1638781

species
301302

SRS058723\_contig\_number\_4291
1

genus
1407607
1

species
1150298

SRS043768\_contig\_number\_30050
1

572511
genus
12


SRS012273\_contig\_number\_33658SRS013965\_contig\_number\_26579SRS022713\_contig\_number\_1060SRS077730\_contig\_number\_18796SRS098655\_contig\_number\_13548SRS103987\_contig\_number\_34760SRS1041091\_contig\_number\_21792SRS1041136\_contig\_number\_9269SRS104485\_contig\_number\_1084SRS146812\_contig\_number\_40409SRS147557\_contig\_number\_7760SRS149244\_contig\_number\_contig-100\_24.81898
12
species
33039

31979
family
1

1
genus
1485

1

SRS148424\_contig\_number\_26155
1896983
species
